# Zika Virus on a Spreading Spree: what we now know that was unknown in the 1950’s

**DOI:** 10.1186/s12985-016-0623-2

**Published:** 2016-10-06

**Authors:** Rupsa Basu, Ebenezer Tumban

**Affiliations:** Department of Biological Sciences, Michigan Technological University, Houghton, MI 49931 USA

**Keywords:** Zika virus, Sexual transmission, Neurological development, Microcephaly, Antibody-dependent enhancement

## Abstract

Zika virus (ZIKV) is a mosquito-borne flavivirus that is transmitted through the bite of *Aedes spp* mosquitoes and less predominantly, through sexual intercourse. Prior to 2007, ZIKV was associated with only sporadic human infections with minimal or no clinical manifestations. Recently the virus has caused disease outbreaks from the Pacific Islands, the Americas, and off the coast of West Africa with approximately 1.62 million people suspected to be infected in more than 60 countries around the globe. The recent ZIKV outbreaks have been associated with guillain-barré syndrome, congenital syndrome (microcephaly, congenital central nervous system anomalies), miscarriages, and even death. This review summarizes the path of ZIKV outbreak within the last decade, highlights three novel modes of ZIKV transmission associated with recent outbreaks, and points to the hallmarks of congenital syndrome. The review concludes with a summary of challenges facing ZIKV research especially the control of ZIKV infection in the wake of most recent data showing that anti-dengue virus antibodies enhance ZIKV infection.

## Highlights

- ZIKV can be transmitted sexually and during pregnancy from a mother to her fetus.
- In addition to microcephaly, ZIKV causes miscarriage.
- ZIKV persists in whole blood for close to 2 months.
- Dengue viral infections enhance ZIKV infections.
- Anti-ZIKV antibodies in domestic animals suggest that ZIKV can infect domestic animals.

## Background

**Fig. 1:**
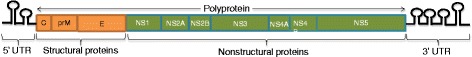
ZIKV genome. The linear genome is made up of structural proteins, nonstructural proteins, and UTRs. The 5’ and 3’ UTRs are ~107 nucleotides and ~428 nucleotides , respectively

Zika virus (ZIKV) is a positive-sense single-stranded RNA virus in the genus *Flavivirus*. The virus is related to other flaviviruses (dengue viruses-DENVs, yellow fever virus-YFV, Japanese encephalitis virus, St. Louis encephalitis virus, West Nile virus, tick-borne encephalitis virus, Langat virus, Powassan virus, Modoc virus, Rio Bravo virus) in terms of genome size and genome organization [1, 2] (Fig. 1). The genome is ~10.7 kb in length and codes for a single polyprotein (~10.2 kb), which is processed into three structural proteins (Capsid-C, pre-Membrane/Membrane-prM/M and Envelope-E) and seven nonstructural proteins (NS1, NS2A, NS2B, NS3, NS4A, NS4B, and NS5) (Fig. 1). The genome is flanked by the 5’ and 3’ untranslated regions (UTRs) [1, 3, 4]. ZIKV has been associated with a lot of disease outbreaks within the last decade. With this in consideration, we searched peer-reviewed articles, government news briefings in Associated Press, and press releases by international organizations for information related to ZIKV. We searched specifically for ZIKV isolation, what was known when the virus was first isolated (clinical manifestations), modes of transmission, the vectors that can transmit the virus, disease outbreaks especially from 2007, and the clinical/pathological manifestations observed in recent outbreaks. The information gathered from these searches was then used to write this review, which summarizes incidences & global distribution of ZIKV infection (within the last decade), modes of transmission, and challenges associated with ZIKV research.

### Epidemiology

**Fig. 2:**
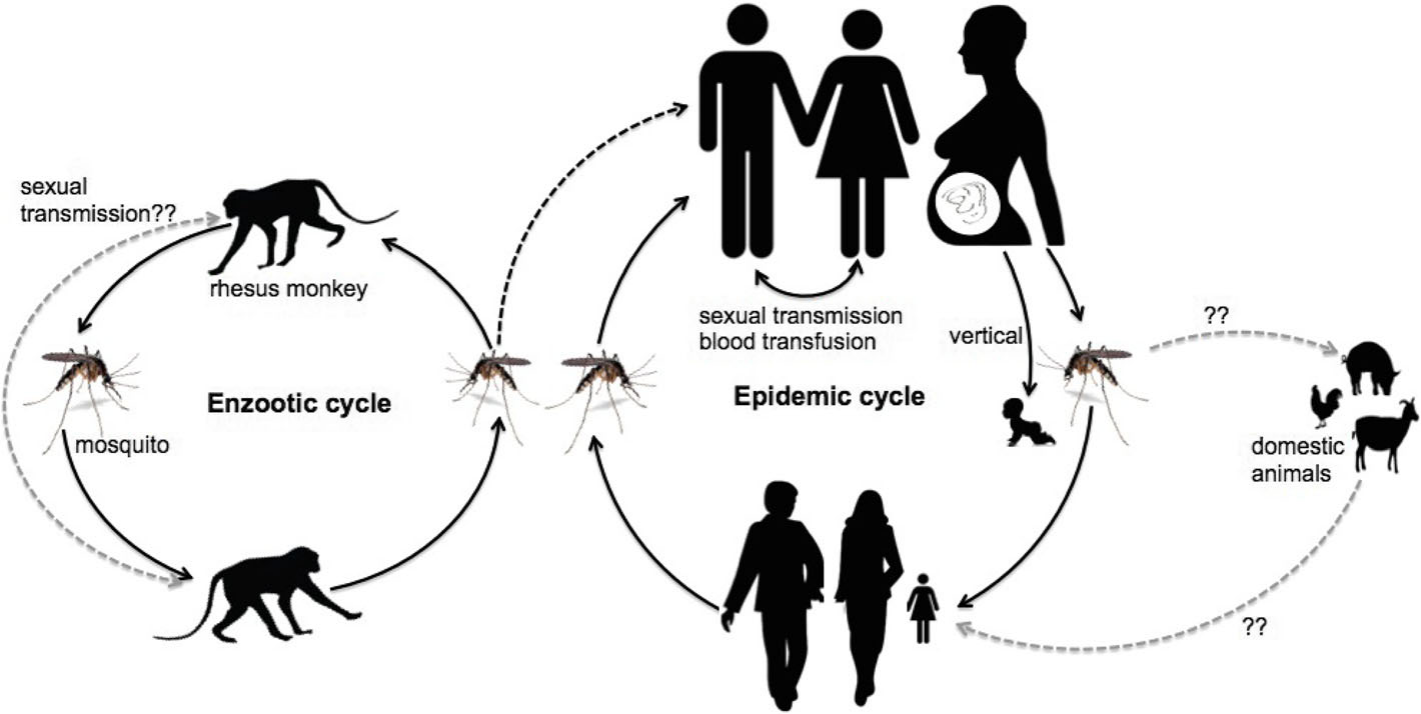
ZIKV transmission cycles. ZIKV is transmitted in sylvatic habitats in an enzootic cycle by infected mosquitoes to rhesus monkeys and vice versa. Humans can be infected with the virus in sylvatic habitats following a mosquito bite or if there is a spillover of an infected mosquito from sylvatic habitats (*middle dotted black line*) to rural/urban areas. An epidemic cycle starts when humans are bitten by an infected mosquito followed by viral replication in humans and viremia. The virus can spread to the reproductive organs and can be transmitted during sexual intercourse. Infected pregnant women can also transmit the virus to the fetus during pregnancy. The virus can then be transmitted from an infected person back to mosquitoes through mosquito bites. The virus then replicates in mosquitoes and it is transmitted back to humans and the cycle continues. It is not known whether the virus can be transmitted by mosquitoes between domestic animals and humans (*right dotted gray lines with question mark*) or whether the virus can be transmitted sexually between monkeys (*left dotted gray line with question mark*)

Although ZIKV is closely related to other flaviviruses in terms of genome size and genome organization, the virus is most closely related to DENVs and YFV in terms of mosquito vector transmission. ZIKV is a mosquito-borne virus that is transmitted primarily through the bite of *Aedes spp* mosquitoes. The virus was first isolated in 1947 from monkeys in Zika forest in Uganda, Africa [5]. Since then, the virus has been isolated from other *Aedes* mosquitoes (*Aedes aegypti*, *Aedes albopictus*, *Aedes africanus*, *Aedes hensilli*, *Aedes polynesiensis*, *Aedes furcifer, Aedes vitattus)* [6] and more recently from a domestic mosquito, *Culex quinquefasciatus* [7]. In sylvatic habitats, ZIKV is transmitted in an enzootic cycle between non-human primates by mosquitoes. In an epidemic cycle, the virus is transmitted between humans primarily by infected mosquitoes [5, 8, 9] (Fig. 2). Introduction of the virus to a human community may be initiated by a spillover mosquito from sylvatic habitats; alternatively, the virus may be imported by humans from countries with ZIKV breakout. Antibodies against ZIKV have been detected in domestic animals such as goats, sheep, rodents [10] but it is unknown whether mosquitoes can transmit the virus between domestic animals or between domestic animals and humans.

Phylogenetically, ZIKV can be divided into two main lineages—African and Asian—based on geographic origin. The African lineage is further sub-divided into West and East African sub-lineages [11]. ZIKV has been associated with a number of sporadic human infections, based on the detection of anti-ZIKV antibodies in serum, starting from 1952 in Africa [12] and 1954 in Asia [13]. In 2007, the virus caused the first major outbreak in the Pacific Islands [14], which later spread to other countries.

- *ZIKV outbreak path since 2007*

**Fig. 3:**
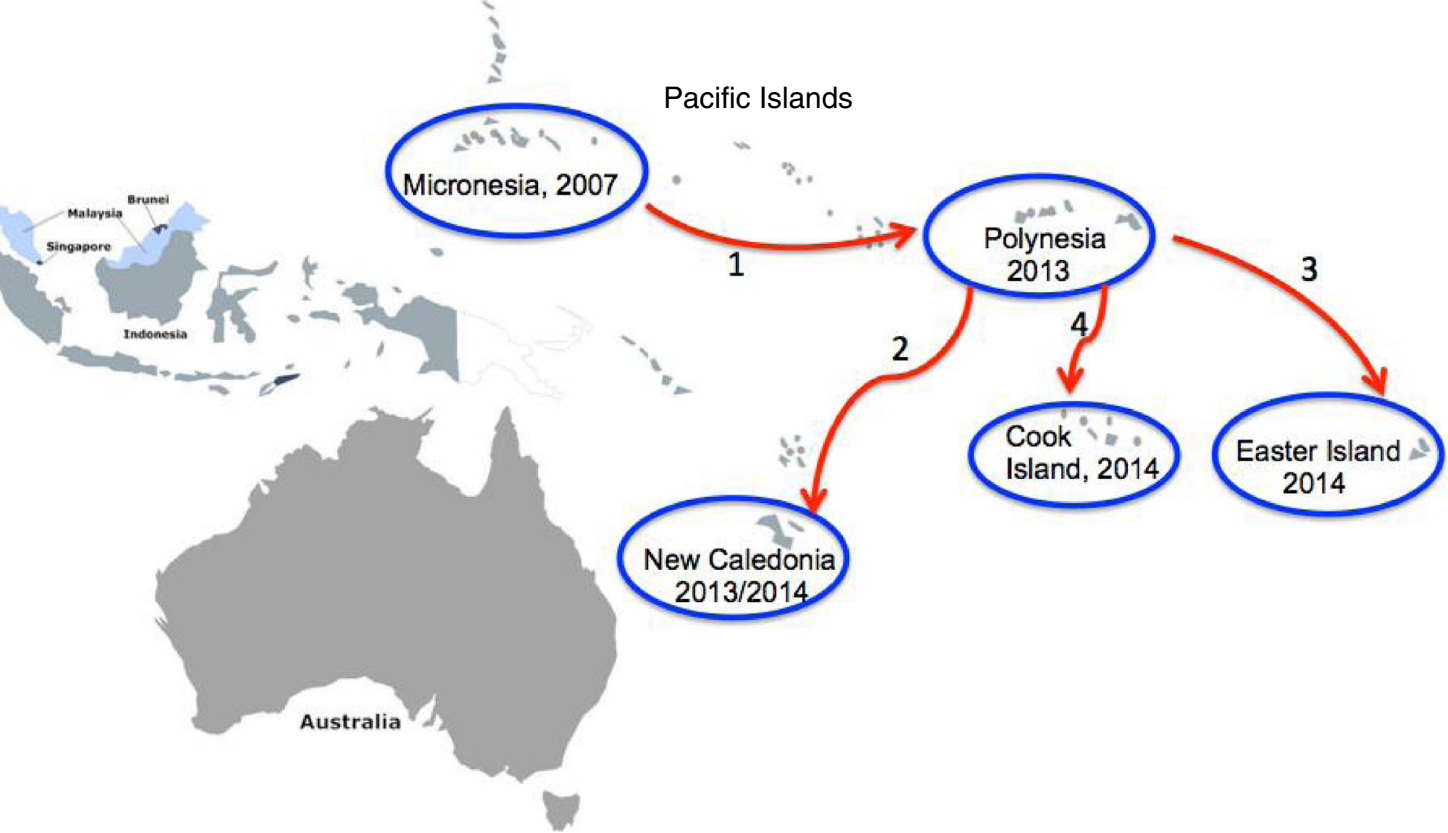
ZIKV outbreaks and transmission paths in the Pacific Islands. The first ZIKV outbreak in the Islands was reported in Yap Island in Micronesia (2007) and it was later transmitted (indicated as red arrow number 1) to French Polynesia in 2013. From French Polynesia, the virus was then transmitted to New Caledonia, Easter Island, and to Cook Island (in the order listed and indicated as red arrow numbers 2, 3, 4)
ii) *The Americas:* The first cases of ZIKV outbreak in Latin America were reported in Brazil (March 2015). Since then, more than 1.5 million people are estimated to have been infected in Brazil alone [23]. Mosquito-transmitted cases of the virus have been reported throughout the Americas (except Canada and Chile) [23–26], as predicted by the World Health Organization (WHO) and Pan American Health Organization (PAHO) (Fig. 4), with more than 65,000 confirmed and suspected cases reported in Colombia alone [27]. Although the number of cases are decreasing in most countries in the Americas and the Caribbean [28], the number of mosquito-transmitted cases are increasing in some countries. For example, as of mid September, more than 85 cases of autochthonous transmissions have been reported in the State of Florida in continental United States [29]. Furthermore, the number of ZIKV imported and sexually transmitted cases continue to increase; more than 3130 imported cases (numbers correct as of mid September) have been reported in the continental United States since the outbreak started in Brazil [30] (Fig. 4). In Cuba, the number of imported cases increased from 1 case in March to 33 cases (3 local transmissions) as of mid September [31]. In summary, an estimated 1.6 million people are suspected to be infected in the Americas.

From 2007, ZIKV outbreaks have been reported in many islands and continents as follows:

i) *Pacific Islands*: In 2007, a ZIKV outbreak from autochthonous transmission was reported in Yap Island in the Federated States of Micronesia with 185 people infected (includes confirmed, probable, and suspected cases) [14]. This outbreak was caused by the Asian lineage of ZIKV. Six years later (in 2013), another outbreak was reported ~5000 miles from Yap Island, in French Polynesia (Fig. 3); more than 28,000 people were infected with ZIKV in this outbreak [15, 16]. The ZIKV strain in the French Polynesia outbreak had 99.9 % nucleotide and amino acid identities with the Asian strain outbreak in the Yap Island [1, 3, 15] suggesting that the French Polynesia outbreak originated from Yap Island. Given the distance between the two Islands, it is unlikely that the virus was introduced into French Polynesia my mosquitoes; this suggests that ZIKV was imported into French Polynesia. The French Polynesia outbreak was subsequently spread to other Pacific Islands. Towards the end of 2013, imported cases from French Polynesia were reported in New Caledonia and cases of autochthonous transmission were reported in January 2014 [17, 18] (Fig. 3). At the same time in January, an outbreak was reported in Easter Island, off the coast of Chile [19] and in February, another outbreak was reported in Cook Islands [17, 18], (Fig. 3). The nucleotide sequence of the ZIKV strain in Easter Island was 99.9 % identical to the ZIKV strain in the outbreak in French Polynesia, thus suggesting that the Easter Island outbreak originated from French Polynesia. Then in March 2015, first cases of ZIKV outbreak (from autochthonous transmission) were reported in Bahia, Brazil. Nucleotide sequence analysis from this outbreak showed 99 % identity with the ZIKV strain that caused the 2013 outbreak in French Polynesia [20] thus suggesting that ZIKV was introduced to the Americas from any of the Pacific Islands (French Polynesia, New Caledonia, Easter Island, or Cook Islands). It is likely that the outbreaks in almost all these islands including the one in Brazil were first imported to these islands/country by an infected individual(s), who later served as reservoir host(s) for mosquito transmission to naïve individuals; ZIKV vectors are endemic in the Pacific Islands and in Brazil [21, 22]. Alternatively, the virus could have been transmitted sexually from an infected traveler to a naïve person in any of these countries.

**Fig. 4:**
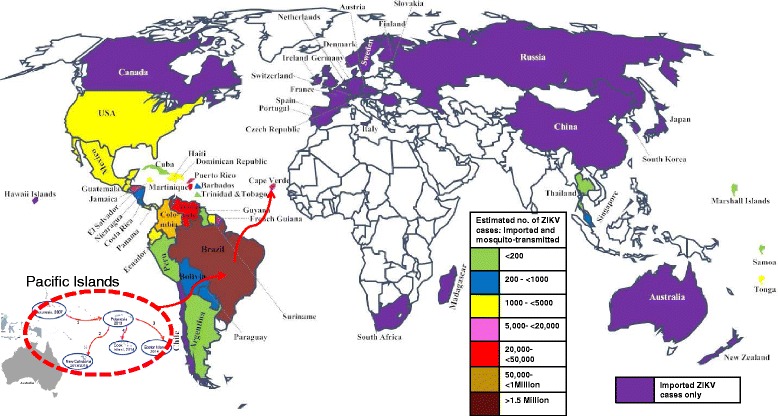
Autochthonous and imported cases of ZIKV around the world from 2015. ZIKV was first introduced to the Americas (Brazil) from the Pacific Island (*indicated as red dotted circle with red arrow*). From Brazil, the virus then spread to countries in South America, Central America, the Caribbean, and off the cost of West Africa (Cape Verde). Countries with only imported ZIKV cases, from the Americas, are shown in purple color. Countries with both mosquito-transmitted and imported cases are shown in different colors with an estimated number of suspected ZIKV cases in the respective countries. Countries with white colors have not reported any imported cases since 2015 (numbers correct as of September)
iii) *Off the cost of West Africa, Cape Verde*: ZIKV outbreak has also been reported in the Islands of Cape Verde, off the coast of West Africa. The first ZIKV cases in the Islands were reported in late September to mid October of 2015 and as of August 2016, more than 7550 people were already infected [32] (Fig. 4). The genetic sequence of the ZIKV strain in Cape Verde has recently been determined and it is identical to the Asian strain in the outbreak in Brazil [32]. The virus was likely imported into the Islands from Brazil by traveler(s) and it was subsequently transmitted from the traveler(s) to naïve individuals through mosquito bites or through sexual contact. Brazil and Cape Verde are close countries (distance-wise) that speak the same language (Portuguese), share almost the same culture and as such, their citizens travel frequently between the two countries. This made it very easy for an asymptomatic ZIKV-infected individual to carry the virus from one country to the other.

It is worth mentioning that since the 2015 outbreak in Brazil, imported cases of ZIKV by travelers from countries with outbreaks have been reported all over the world.

b) *Imported cases around the world*:

In North America, the number of ZIKV imported cases from Latin America continue to increase. As of mid September 2016, the number of imported ZIKV infections reported in Canada were more than 279 [33] (Fig. 4). The number of imported cases are also increasing in Europe; the number of imported cases increased from 224 in March 2016 to 1265 cases in August 2016. The highest number of imported cases have been reported in France and Spain [34]. In Eurasia/Asia, 6 imported cases have been reported in Russia and more than 21 cases have been reported in China [35, 36]. In Singapore and Thailand, more than 300 and 200 cases have been reported, respectively (numbers correct as of mid September) [37]. In Africa, 1 imported case from Columbia has been reported in South Africa [38]. In-between the Pacific and Indian Oceans, 12 and >44 imported cases have been reported in Hawaii [30] and Australia [39], respectively. Thus, the virus has been imported to at least one country in every continent except Antarctica, making this the first ZIKV pandemic the world has ever experienced.

### Transmission

ZIKV was believed (until less than a decade ago) to be transmitted to humans only through the bites of *Aedes spp* mosquitoes. Recently, other modes of human transmission have been documented as follows:

i) *Sexual transmission*. ZIKV has been detected in the urine and semen of ZIKV-infected patients [40, 41] with more than 30 cases of sexual transmission from male to female, 1 case from male to male and 1 from female to a male have been reported [42–47]. This observation shows that the virus can be transmitted between both sexes but the highest frequency of transmission is from male to female.
ii) *Vertical transmission from mother to fetus*. ZIKV has been detected in amniotic fluid, fetal brain, and also in the serum of babies, 4 days after birth [48–51], thus demonstrating that the virus can be transmitted to the fetus during pregnancy (Fig. 2).
iii) Blood transfusion. Two cases of ZIKV transmission by blood transfusion have been reported in Brazil [52]. This observation will make ZIKV transmission more complicated given the fact that a majority of ZIKV-infected patients do not show symptoms; in fact, 3 % of ZIKV asymptomatic blood donors have tested positive for ZIKV [53]. This makes it very easy for the virus to be transmitted from blood donors to blood recipients. The situation is also exacerbated by the fact that the virus can persists in whole blood of patients for close to 2 months [46, 54].

### Clinical manifestations

**Fig. 5:**
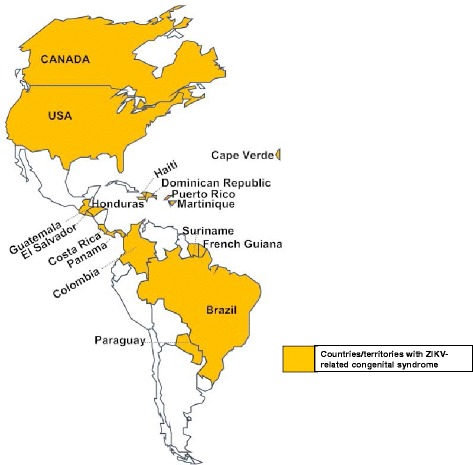
Countries and territories with cases of ZIKV-related congenital syndrome (microcephaly or congenital central nervous system anomalies). ZIKV-related congenital syndrome cases have been reported in Brazil, Colombia, Cape Verde, Martinique, Panama, El Salvador, Paraguay, French Guiana, Puerto Rico, Canada, the United States, Costa Rica, Guatemala, Honduras, Dominican Republic, Haiti, and Suriname

When anti-ZIKV antibodies were first detected in human sera in the early 1950s, the authors pointed out that “The effects of this agent in man are quite unknown” [13]. The reason they made this statement was due to the fact that the population sampled at that time did not show any clinical or pathological manifestations, that could be associated with ZIKV infection. As mentioned above, most people infected with ZIKV are asymptomatic. However, 20–25 % of infected patients develop symptoms such as fever, skin rash, joint pains, headache, and conjunctivitis within 1 week after infection; in addition to this, some patients experience hematospermia [43, 46, 55]. Although ZIKV infection is not life threatening in healthy adults, the virus can cause the following debilitating conditions:

i) *Neurological problems such as guillain-barré syndrome (GBS; an autoimmune disease)* [56]. Ninety-eight to a hundred % of patients diagnosed with GBS during the French Polynesia ZIKV outbreak had anti-ZIKV antibodies, compared to 56 % of patients without GBS [57]. The mechanism(s) underlying the contribution of these anti-ZIKV antibodies to GBS is still unknown.
ii) *Miscarriage and congenital syndrome such as microcephaly (a neurodevelopmental disorder whereby babies are born with an abnormally small head) or an abnormally developed congenital central nervous system* [48, 56, 58, 59]. ZIKV infects a population of developing brain cells including embryonic forebrain-specific human neural progenitor cells, neurospheres and brain organoids thus causing increased cell death, cell cycle dysregulation and ultimately reduced cell growth [60, 61]. These developmental changes are probably the hallmarks of congenital syndrome. In fact, these observations may explain the reason behind the increase in the number of congenital syndrome cases reported in ZIKV-infected countries in the Americas and Cape Verde; more than 16 countries have reported cases of ZIKV-related congenital syndrome (Fig. 5). In Brazil alone, 1911 cases have been confirmed with 371 neonatal deaths reported; in Columbia and Cape Verde, 40 and 14 cases, respectively, of ZIKV-related congenital syndrome have also been reported (numbers correct as of September) [31, 62]. Additionally, cases of ZIKV-related miscarriages have been reported in other countries [51, 59]. These devastating effects have prompted many countries to advise pregnant women to avoid visiting regions (most recently, Florida in the US, Singapore and Thailand in Asia) with ZIKV outbreaks [42, 63]. Overall, ZIKV infection seems to have the highest morbidity in newborn infants.

### Challenges associated with ZIKV infections

i) *There are no vaccines to protect against ZIKV infection or drugs to treat infected patients.* The majority of ZIKV infected patients recover from the infection and do not need treatment. However, as mentioned above, transmission of the virus from pregnant women to fetuses affects normal fetal neurological developments. As such, women, especially those who plan to be become pregnant, need to be immunized. Thus, there is an urgent need to develop a vaccine to stop the spread of ZIKV infection, especially from pregnant women to fetuses. The development of an effective ZIKV vaccine will be challenging for the following reasons:
  - although 9 % of anti-dengue virus monoclonal antibodies can cross-neutralize ZIKV infections, a majority of anti-dengue virus antibodies are not neutralizing [64]; instead, they enhance ZIKV and dengue viral infections, a condition known as antibody-dependent enhancement of infection [65, 66]. This observation may complicate ZIKV infections in countries were ZIKV and dengue viral infections co-circulate and especially in countries (Brazil, Mexico, El Salvador, and the Philippines) where dengue virus vaccine (dengvaxia) has been licensed; the effect of the vaccine on ZIKV infection needs to be evaluated. With this in mind, an ideal ZIKV vaccine should not enhance dengue infection and vice versa.
  - a candidate ZIKV vaccine should protect against all ZIKV strains. Additionally, it should lack the propensity to be accidentally transmitted by mosquitoes from vaccinees to unvaccinated population. This will be a big challenge for an attenuated ZIKV vaccine given the fact that the virus is transmitted by mosquito-bites.
  - the vaccine should be safe in pregnant women in order to avoid complications during pregnancy.
  - an effective ZIKV vaccine should elicit a systemic immune response in addition to genital immunity given the fact that the virus can also be transmitted sexually. A ZIKV vaccine that cannot protect against sexual transmission may not be highly valuable.

ii) *ZIKV is transmitted by some of the same Aedes mosquitoes (e.g. Aedes aegypti) that transmit DENVs, YFV, and Chikungunya virus (an alphavirus)* [18, 67]*.* To aggravate the situation, the symptoms (fever, skin rash, joint pains, and headache) for ZIKV infection are similar to those caused by these three viruses. As such, most ZIKV infections are clinically misdiagnosed as DENV infections.
iii) *Serological tests for ZIKV targeting the envelope glycoprotein domains, EI and EII, are not specific*; they cross-react with other flaviviruses such as DENVs and YFV [1, 4, 65, 68]. These domain-cross-reactive antibodies can misdiagnose ZIKV infections as dengue and vice versa.
iv) *ZIKV RNA/viral particles have been isolated or detected in nasopharynx* [69]*, saliva* [49, 70, 71]*, and in breast milk* [49]. Nevertheless, it is not known if the virus can be transmitted through saliva, nasal secretions, or breast milk. Moreover, it is unknown if anti-ZIKV antibodies are present in these secretions. Studies are needed to assess if the virus can be transmitted through these routes and if anti-ZIKV antibodies are present in body secretions such as saliva and breast milk.
v) *Although ZIKV RNA and viral particles have been isolated from saliva, non-invasive methods using saliva to diagnose ZIKV are lacking*. As such, there is need for rapid diagnostic kits for detecting ZIKV infection using saliva.
vi) *Although we know that ZIKV can be transmitted sexually from male to female, male to male, and female to male, there has been no report on transmission from female to female couples.* Studies are required to assess transmission between female-sex partners.
vii) Recent isolation of ZIKV infection from *Culex quinquefasciatus mosquitoes* [7] *may also make the control of ZIKV infection challenging. Culex quinquefasciatus* just like *Aedes* mosquitoes is a domesticated mosquito that breeds in standing water, is widespread in the Americas (except Canada), Africa, Asia, the United Kingdom, and Pacific Islands [72], and feeds on humans, domestic animals, including birds [73]. Thus, measures to control *Aedes* mosquitoes also have to take the control of *Culex quinquefasciatus* mosquitoes into consideration.
viii) *It is unknown why some patients (65 %) with anti-ZIKV antibodies do not develop GBS whereas some patients do*. Does the genetic make-up of an individual pre-dispose that individual to ZIKV-associated GBS or microcephaly?

## Conclusions

ZIKV infection is a major public health problem that has already spread to many countries around the globe, and is likely to spread to more given the fact that the virus can be transmitted sexually and by mosquitoes to humans. Imported ZIKV by asymptomatic travelers will likely be transmitted to sexual partners thus increasing the number of infected people and consequently the availability of ZIKV-infected blood meal for naïve mosquitoes. A blood meal from infected patients, following a mosquito bite, will be all it takes to establish an outbreak in countries with imported cases, as has been demonstrated in the United States. This observation puts China, Singapore, Thailand and Europe, which all have *Aedes aegypti* and *Aedes albopictus* mosquitoes, at high risk for mosquito transmission given the fact that ZIKV has already been imported to these regions [21, 22, 37]. An infected mosquito in any of these regions can cross, irrespective of country borders, from one country to another thus increasing the spread of the virus. It is also likely that people moving across country borders will spread the virus to other countries.

We now know, unlike in the 1950s, that ZIKV cannot only be transmitted through mosquito bites; it can also be transmitted sexually, via blood transfusion, and from mother to fetus. We also know that the virus is associated with symptoms such as joint pains, skin rash, and that it causes neurological problems such as GBS, microcephaly and abnormally developed congenital central nervous system. Nevertheless, there are a lot of things we still do not know about ZIKV; we do not know whether the virus can be transmitted through nasal secretions, saliva or breast milk. Also, there are still unanswered questions as to why the outbreak in Brazil spread to almost all of the Americas and why this outbreak had a higher mortality and morbidity compared to sporadic outbreaks prior to 2007. With these in consideration, there is an urgent need to develop vaccines and therapeutics to prevent or stop the spread of ZIKV infections. In addition to this, there is an urgent need to develop robust diagnostic tests that can detect and discriminate ZIKV infections from other flaviviruses (DENVs, Chikungunya, and YFV), 5 days after onset of symptoms. Fortunately, recent studies have shown that antibodies targeting NS1 proteins are ZIKV-specific and can be used to develop ZIKV-specific diagnostic kits [65]. If an antigen diagnostic test targeting ZIKV NS1 is successfully developed, it can be used to diagnose ZIKV infections from the onset of viremia. In fact, a ZIKV antigen diagnostic test will be more valuable because they can detect ZIKV infections prior to the appearance of anti-ZIKV antibodies in serum. Such a test can be used along-side with a specific molecular test, such as reverse transcription (RT) PCR, to confirm ZIKV infection. Until then, specific detection of ZIKV infections has to rely on RT-PCR.
